# Assembly of Iron Oxide Nanocubes for Enhanced Cancer Hyperthermia and Magnetic Resonance Imaging

**DOI:** 10.3390/nano7040072

**Published:** 2017-03-28

**Authors:** Minjung Cho, Antonio Cervadoro, Maricela R. Ramirez, Cinzia Stigliano, Audrius Brazdeikis, Vicki L. Colvin, Pierluigi Civera, Jaehong Key, Paolo Decuzzi

**Affiliations:** 1 Department of Translational Imaging & Department of Nanomedicine, Houston Methodist Research Institute, Houston, TX 77030, USA; supia1001@gmail.com (M.C.); MReyes2@houstonmethodist.org (M.R.R.); cinzia.stigliano@gmail.com (C.S.); 2 Department of Chemistry, Rice University, Houston, TX 77005, USA; vicki_colvin@brown.edu; 3 Laboratory of Nanotechnology for Precision Medicine, Fondazione Istituto Italiano di Tecnologia, 16163 Genova, Italy; Antonio.Cervadoro@iit.it; 4 Department of Physics and Texas Center for Superconductivity, University of Houston, Houston, TX 77004, USA; audrius@uh.edu; 5 Dipartimento di Elettronica e Telecomunicazioni, Politecnico di Torino, I-10129 Torino, Italy; keyjae@gmail.com; 6 Department of Biomedical Engineering, Yonsei University, 1 Yonseidae-gil, Wonju, Gangwon-do 220-710, Korea

**Keywords:** nanomedicine, magnetic resonance imaging, specific absorption rate, cancer theranostics, magnetic dragging

## Abstract

Multiple formulations of iron oxide nanoparticles (IONPs) have been proposed for enhancing contrast in magnetic resonance imaging (MRI) and for increasing efficacy in thermal ablation therapies. However, insufficient accumulation at the disease site and low magnetic performance hamper the clinical application of IONPs. Here, 20 nm iron oxide nanocubes were assembled into larger nanoconstructs externally stabilized by a serum albumin coating. The resulting assemblies of nanocubes (ANCs) had an average diameter of 100 nm and exhibited transverse relaxivity (*r*_2_ = 678.9 ± 29.0 mM^‒1^·s^‒1^ at 1.41 T) and heating efficiency (specific absorption rate of 109.8 ± 12.8 W·g^‒1^ at 512 kHz and 10 kA·m^‒1^). In mice bearing glioblastoma multiforme tumors, Cy5.5-labeled ANCs allowed visualization of malignant masses via both near infrared fluorescent and magnetic resonance imaging. Also, upon systemic administration of ANCs (5 mg_Fe_·kg^‒1^), 30 min of daily exposure to alternating magnetic fields for three consecutive days was sufficient to halt tumor progression. This study demonstrates that intravascular administration of ANCs can effectively visualize and treat neoplastic masses.

## 1. Introduction

Iron oxide nanoparticles (IONPs) have been proposed as agents for magnetic resonance imaging (MRI), localized hyperthermia treatment, controlled drug release, and magnetic guidance and manipulation [1,2,3,4,5,6]. IONPs with a characteristic core size smaller than ~30 nm exhibit high transverse relaxivity (*r*_2_), significantly shortening the relaxation time of the surrounding water molecules [7]. Also, localized hyperthermia and tissue thermal ablation can be achieved by exposing IONPs to alternating magnetic fields (AMFs) for sufficiently long periods of time [8,9,10]. Furthermore, static magnetic fields can be used to remotely guide IONPs to specific biological targets and to non-invasively manipulate molecules and cells [11]. Importantly, several reports have shown that IONPs are biodegradable, and that the dissolved iron can participate in the physiological metabolism of cells, thus limiting possible toxicity concerns and supporting repetitive use [12,13]. Their intrinsic theranostic properties, biocompatibility, and biodegradability have contributed to the popularity and success of IONPs in biomedical applications.

These findings have stimulated multiple investigations for improving the performance of IONPs, specifically, their MRI performance and magnetic heating efficiency. One of the strategies to enhance transverse relaxivity *r*_2_ has been to combine individual IONPs into larger polymeric or silica matrices to form multicore assemblies [14,15,16,17]. Among several others, Simard and colleagues encapsulated 10 nm IONPs in pH-responsive hydrogel matrices demonstrating transverse relaxivities as high as 500 mM^‒1^·s^‒1^ at 3 T [15]. In the field of magnetic hyperthermia, most of the efforts have focused on changing the IONP core size, shape, or material composition to increase the saturation magnetization (*M*_s_) and the magnetic anisotropy (*K*) [6,8,18,19]. Among the many tested configurations, nanoparticles synthesized in a cubic shape have been demonstrated to have the best performance by far. Hyeon and his group demonstrated the synthesis of 22 nm ferrimagnetic nanocubes (NCs) coated with polyethylene glycol-phospholipid, which attained an *r*_2_ of 761 mM^‒1^·s^‒^^1^ at 3 T [20,21]. In another paper, the same group reported that 30 nm chitosan oligosaccharide-stabilized ferrimagnetic NCs—i.e., a different NC formulation—could provide a specific absorption rate (SAR) of 2614 W·g^‒1^ at 1 MHz and 0.660 kA·m^‒1^ [22]. The research group of Pellegrino and Gazeau showed that 19 nm water-soluble NCs could provide an SAR of 2452 W·g^‒1^ at 520 kHz and 29 kA·m^‒1^ [18]. Cheon and collaborators developed exchange-coupled core-shell magnetic nanoparticles and nanocubes that also exhibited superior magnetic properties [19]. However, the identification of an optimal iron oxide size range for magnetic thermal therapies via systemic injection is still a matter of intense scientific debate [8,23]. One definite point is that IONPs larger than ~30 nm tend to manifest ferromagnetic behavior, leading to poor colloidal stability, which hampers their biomedical applicability. However, such small magnetic volumes dramatically limit their magnetic guidance efficiency within the blood stream [24].

In this work, NCs were produced via thermal decomposition methods and then assembled into larger nanoconstructs (ANCs), comprising a few NCs coated with double oleic acid or serum albumin layers. The physico-chemical and magnetic properties of the NCs and ANCs were characterized by deriving their MRI relaxivities (*r*_1_ and *r*_2_) and SAR values. In vitro cytotoxicity was assessed in different cell lines. Biodistribution and tumor accumulation under magnetic guidance were studied in a mouse model of glioblastoma multiforme. In vivo magnetic resonance (MR) and near-infrared imaging as well as tumor hyperthermia treatments were performed in the same mouse model of cancer.

## 2. Results

### 2.1. Synthesis and Physico-Chemical Characterization of Assemblies of NCs

Individual and assembled iron oxide nanocubes (NCs) with different core sizes in hexane, ranging from about 10 to 30 nm, were synthesized via high-temperature thermal decomposition methods [20,25] (Supplimentary Figure S1). X-ray diffraction (XRD) patterns and high-resolution TEM (HR-TEM) images showed that the assembled NCs had an inverse cubic spinel phase with magnetite (Fe_3_O_4_), while the individual NCs showed a magnetite phase as well as wüstite (FeO) (Supplementary Figures S2 and S3).

For in vivo application, we modified the surfaces of the individual and assembled NCs with either double oleic acid or bovine serum albumin, and then the surface-modified NCs were analyzed by scanning electron microscopy (SEM), as shown in Supplementary Figure S4, and zeta-potential analysis, as shown in Supplimentary Figure S7. The r_1_ and r_2_ relaxivities and specific absorption rates (SAR) of the individual and assembled NCs were compared (Supplimentary Figures S5 and S6). Among the various NCs, 20 nm assembled NCs showed the highest r_2_ relaxivity (678 mM^‒1^·s^‒1^) and SAR value (109.8 W·g^‒1^) and so were used for further investigation in this study.

**Figure 1:**
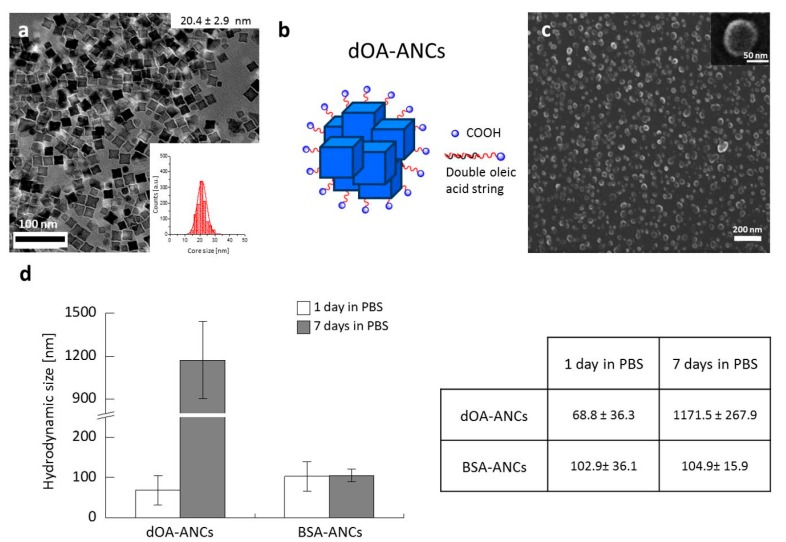
Water-soluble assemblies of iron oxide nanocubes (ANCs). (**a**) Transmission electron microscopy images of 20 nm ANCs in hexanes; inset provides the size distribution of 20.4 ± 2.9 nm NCs; Double oleic acid layer (dOA)-coated ANCs (dOA-ANCs): (**b**) schematic representations and (**c**) scanning electron microscopy images of dOA-ANCs with an average diameter of 67.5 ± 9.4 nm; (**d**) Stability of ANCs with different surface coatings: hydrodynamic diameter stability in phosphate buffered saline (PBS) after one and seven days of ANCs coated with a double oleic acid layer (dOA-ANCs) or bovine serum albumin (BSA-ANCs).

A TEM image of the assembled 20 nm NCs is shown in Figure 1a. After synthesis, the surfaces of the assembled NCs were further modified using either a double-layer coating of oleic acid (dOA-ANCs) [26] or a bovine serum albumin (BSA) coating (BSA-ANCs). A schematic representation of dOA-ANCs is shown in Figure 1b. Scanning electron microscopy (SEM) images of dOA-ANCs and BSA-ANCs are provided in Figure 1c and Supplementary Figure S4, respectively, demonstrating their overall characteristic assembly size of about 100 nm. The stabilities of these two differently coated ANCs were tested in phosphate buffered saline (PBS) and DI water, as documented in Figure 1c and Supplementary Figure S8. The BSA-ANCs showed excellent stability with an average hydrodynamic size of ~100 nm, which was stable for up to seven days. In contrast, the dOA-ANCs showed an initially lower diameter that dramatically increased to ~1200 nm after seven days, demonstrating a tendency to aggregate over time, as shown in Figure 1d. Zeta-potential data of the assembled NCs are plotted in Supplementary Figure S7; the surface charge of the BSA-ANCs was about −20 mV compared with −60 mV for the dOA-ANCs. Note that, of the four different edge lengths considered for the individual NCs (i.e., 11 ± 1, 15 ± 2, 20 ± 3, and 26 ± 3 nm), the assembled 20 nm NCs were by far the best performing in terms of *r*_2_ relaxivity and SAR (Supplementary Figure S5); therefore, 20 nm assembled NCs were generated for subsequent experiments.

### 2.2. MRI Relaxivity and Magnetic Hyperthermia

**Figure 2:**
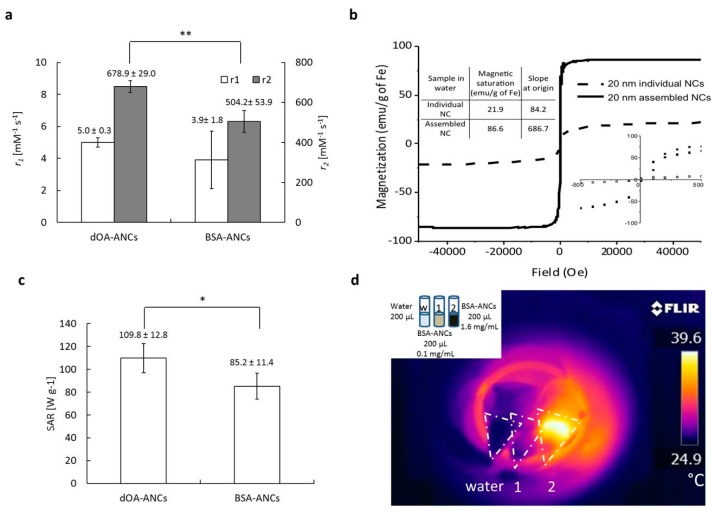
Magnet-related properties of 20 nm ANCs with different surface coatings (double oleic acid [dOA] and bovine serum albumin [BSA]). (**a**) MRI relaxivity (*r*_1_ and *r*_2_) values; (**b**) M–H curve with a magnified view and saturation magnetization values for the individual and dOA-assembled NCs at 300 K; (**c**) specific absorption rates measured at a field strength of 10 kA·m^‒1^ and a frequency of 512 kHz. (N.S., not significant at *p* > 0.1; *, significant at *p* < 0.1; **, significant at *p* < 0.05); (**d**) Temperature responses with water, BSA-CNCs at 0.1 mg·mL^‒1^, and BSA-CNCs at 1.6 mg·mL^‒1^ upon generating alternative magnetic fields. Temperature map generated by a thermo-sensitive infrared camera.

The magnetic properties of the assemblies of NCs were assessed in terms of longitudinal *r*_1_ and transverse *r*_2_ magnetic relaxivities, measured with a bench-top relaxometer (1.41 T), and SAR for both coating configurations, as presented in Figure 2. The longitudinal relaxivities *r*_1_ were comparable and ranged between 4 and 5 mM^‒1^·s^‒1^. However, the transverse relaxivities *r*_2_ of the BSA-ANCs (~500 mM^‒^^1^·s^‒1^) were about 15% lower than those of the dOA-ANCs (~680 mM^‒1^·s^‒1^), as shown in Figure 2a. Nonetheless, these are close to the highest *r*_2_ values documented in the literature [21]. The slightly lower *r*_2_ values measured for the BSA-ANCs could be explained by the difference in diffusion of water molecules within the dOA coating (molecular weight of ~1 kDa) and the albumin coating (molecular weight ~67 kDa). Note that this could also explain the higher stability of the BSA-ANCs compared to the dOA-ANCs. It is also interesting to note that the ANCs exhibited a magnetization saturation close to 90 emu·g^‒1^ (86.6 emu·g^‒1^). The magnetization curve in Figure 2b was plotted at 300 K, showing very low remanence and coercivity by ANCs near the zero field. Also, considering that the remanence and coercivity are affected by the measurement temperature [27], the ANCs can be in the transition states of superparamagnetism and ferrimagnetism. Thus, the heating effect caused by ANCs can be explained by the resonance with Neél and/or Brown motions [28]. Moreover, considering that the Curie temperature (*T*_c_) of magnetic nanoparticles is 42–46 °C (315–319 K) for hyperthermia [29], above this temperature, the magnetic nanoparticles can lose their magnetization. Thus, the mild hyperthermia effect demonstrated by the ANCs in Figure 2d might be effective to continuously damage cancer cells while maintaining the magnetic heating effects. For the SAR values, the BSA-ANCs (85.2 ± 11.4 W·g^‒1^) showed a minor 20% decrease in SAR compared to that of the dOA-ANCs (109.8 ± 12.8 W·g^‒1^) (Figure 2c). Using an infrared camera (FTIR A325), the temperature field generated by the ANC solutions exposed to an AMF was readily depicted (Figure 2d). Two different solutions were used, namely 0.1 and 1.6 mg_Fe_·mL^‒1^, showing the increase in temperature with iron concentration.

### 2.3. Cell Cytotoxicity of ANCs

**Figure 3:**
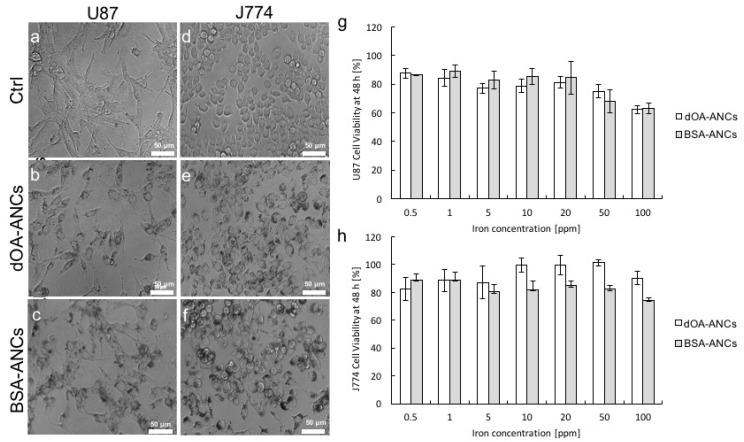
In vitro cell viability with ANCs. Cell internalization and viability studies were conducted on U87-MG glioblastoma multiforme cells and J774 murine macrophages. Bright field images of U87-MG cells incubated with (**a**) no ANCs (ctrl); (**b**) 20 ppm double oleic acid layer (dOA)-coated ANCs (dOA-ANCs); and (**c**) 20 ppm bovine serum albumin-coated ANCs (BSA-ANCs). Bright field images of J774 macrophages incubated with (**d**) no ANCs (ctrl); (**e**) 20 ppm dOA-ANCs; and (**f**) 20 ppm BSA-ANCs; Cell viability tests on (**g**) U87-MG cells and (**h**) J774 macrophages incubated with differently coated ANCs for up to 48 h.

Before we proceeded with our in vivo experiments, the cell cytotoxicities of the ANCs with dOA and BSA coatings were assessed using a 3-(4,5-dimethylthiazol-2-yl)-2,5-diphenyltetrazolium bromide (MTT) assay. Two different cell lines were considered, namely U87-MG cells of human glioblastoma multiforme tumors and J774 murine macrophages (Figure 3). Different concentrations of ANCs with dOA or BSA coatings, ranging from 0.5 to 100 ppm, were incubated with these cells for 48 h. Figure 3 compares bright field microscopy images of cells incubated with 20 ppm_Fe_ of ANCs coated with dOA or BSA. The U87-MG cells incubated for 48 h with different concentrations of ANCs showed 80% viability with ANC concentrations up to 20 ppm_Fe_. At 100 ppm_Fe_, the viability of the U87-MG cells decreased to about 60% (Figure 3g). Similarly, the J774 macrophages demonstrated no significant toxicity up to 100 ppm_Fe_ (Figure 3h). From this, it can be concluded that ANC suspensions with dOA or BSA coating did not exhibit a significant toxic effect on these cell lines.

### 2.4. Biodistribution Near Infrared Fluorescence and MRI

**Figure 4:**
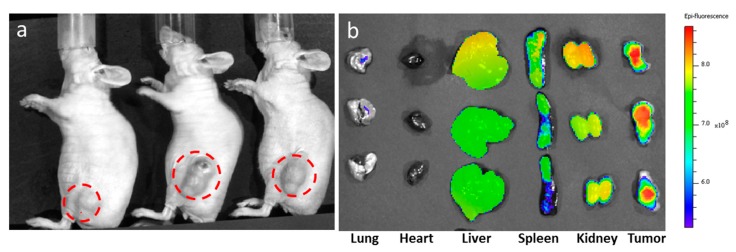
Near infrared fluorescence imaging and biodistribution. ANCs were coated with bovine serum albumin and labeled with the near infrared dye Cy5.5 (Cy5.5-ANCs). Then, 0.25 mg_Fe_·kg^‒1^ of Cy5.5-ANCs were injected via a tail vein, and a static magnet was positioned next to the tumor for up to 40 min post-injection. Fluorescent images of mice bearing U87-MG tumors: (**a**) cells before injection (*n* = 3) and (**b**) ex vivo fluorescent images of different organs harvested at 24 h post-injection.

Due to their higher stability over time, the BSA-ANCs were selected for animal studies. In the biodistribution tests, BSA-ANCs were also labeled with a near infrared dye (Cy5.5). The resulting near infrared fluorescence (NIRF) nanoconstructs (Cy5.5-ANCs) were used in a mouse model of glioblastoma multiforme (*n* = 3). Figure 4 shows the biodistribution of the Cy5.5-ANCs. Following the systemic injection of 0.25 mg_Fe_·kg^‒1^, a static magnet was applied to the tumor area (indicated by red dashed circles) for 40 min post-injection (p.i.) to enhance tumor accumulation (Figure 4a). The ex vivo images in Figure 4 were taken at 24 h p.i. for the harvested organs. Although fluorescent signals were detected in the liver, spleen, and kidneys, a higher fluorescent intensity was clearly associated with the malignant mass at 24 h p.i. (Figure 4b).

**Figure 5:**
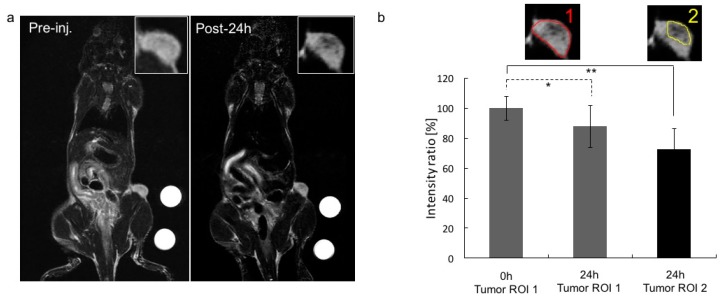
In vivo MR imaging. In this trial, 5 mg_Fe_·kg^−1^ BSA-ANCs were intravenously injected into mice bearing U87-MG tumor cells (*n* = 3). No magnets were used to enhance the tumor accumulation of ANCs at this time. (**a**) MR images were taken before (left) and 24 h after (right) the injection of the BSA-ANCs via a tail vein. A 3T clinical MRI scanner was used; (**b**) Intensity ratios for two regions of interest (ROIs), as depicted in the insets, at 0 and 24 h post-injection. The intensity ratios were calculated by averaging the MRI signal over multiple z-planes of the malignant mass. N.S., not significant at *p* > 0.1; *, significant at *p* < 0.1; **, significant at *p* < 0.05.

Similar experiments were performed to characterize the in vivo MRI performance of the BSA-ANCs. A 3T clinical scanner (Philips Ingenia) equipped with a mouse coil was used for these characterizations after tail vein injection of 5 mg_Fe_·kg^‒1^ animal (*n* = 3). Note that no external magnet was used in this case. Figure 5a shows T_2_-weighted MR images of one representative mouse bearing U87-MG cells in the flank, before ANC injection (left) and at 24 h p.i. (right). In a comparison of the two insets (top, right), the malignant mass appears darker (signal attenuation) at the latest time point, demonstrating the accumulation of T_2_ contrast-enhancing agents. Figure 5b quantifies the change in contrast in two different regions of interest (ROIs), as depicted in the insets. The signal attenuation was significant for both ROIs at 24 h p.i. The NIRF and MR images in Figure 4 and Figure 5, respectively, demonstrate the tumor accumulation of BSA-ANCs within 24 h post-injection.

### 2.5. In Vivo Magnetic Hyperthermia

**Figure 6:**
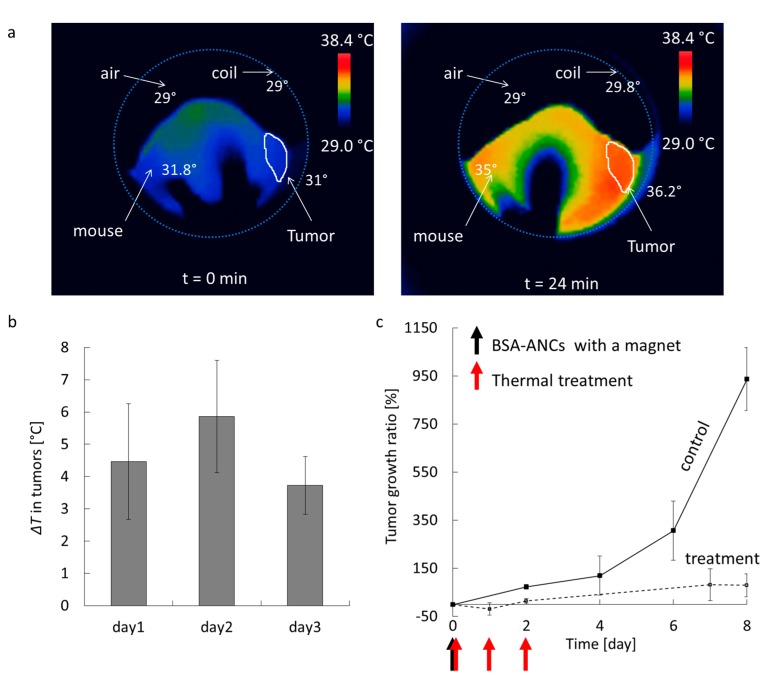
In vivo magnetic hyperthermia. Here, 5 mg_Fe_·kg^‒1^ of BSA-ANCs were systemically injected into mice bearing U87-MG tumor cells (*n* = 3). A magnet was kept next to the tumor mass for up to 40 min post-injection. (**a**) Temperature map before and at 30 min after treatment initiation showing the experimental set up with the coils (light blue circle) and the mouse lying within. The tumor is shown in the white region on the right side of the animal; (**b**) Average temperature increase for the tumor over the three cycles of hyperthermia (days 1, 2, and 3) after systemic injection of BSA-ANCs; (**c**) Tumor growth ratio, defined as (*V* − *V*_0_)/*V*_0_ × 100, where *V* is the tumor volume, and *V*_0_ is the tumor volume at time zero.

The therapeutic activity of BSA-ANCs was assessed in mouse models (*n* = 3) of glioblastoma multiforme. First, 5 mg_Fe_·kg^‒1^ were injected via a tail vein, and a magnet was applied to the tumor area for 40 min p.i. to enhance tumor accumulation. Figure 6a shows infrared images of a representative mouse, lying within the coils of the AMF generator, before (left) and at 30 min after initiation (right) of the treatment. Before starting the experiment, the coils looked dark, and the overall mouse body temperature was ~31 °C, whereas the tumor mass on the right side appeared at a slightly lower temperature of ~28 °C. Upon activation of the AMF generator (field strength: 10 kA·m^‒1^, frequency: 512 kHz), the tumor temperature increased by ~5 °C within 30 min, as shown in Figure 6b, for each consecutive treatment. Please note that infrared thermometry is a surface measurement; a further increment of about 3 °C is expected to be achieved inside the tumor. Moreover, as compared with this heating effect with/without ANCs, it was clear that this change was caused only by ANCs with AMF (Supplementary Figure S9). With this increased temperature, changes in enzymatic activities can affect cell structures and cell growth or differentiation, inducing cell apoptosis and/or necrosis [30]. The AMF exposure of the malignant mass for 30 min for three consecutive days post BSA-ANC injection was sufficient to dramatically reduce the tumor growth ratio. This is shown in Figure 6c, demonstrating a significant difference in tumor growth between the treated mice and the untreated control animals. Notably, the untreated mice experienced an almost 10-fold increase in tumor size over eight days, whereas the animals injected with BSA-ANCs and exposed to AMF had only a moderate 0.5-fold increase in tumor mass within the same time period.

## 3. Discussion

Iron oxide nanocubes were synthesized and characterized for their magnetic properties, including transverse relaxivity *r*_2_ and specific absorption rate. Among four different cube lengths, 20 nm NCs manifested the highest values for both transverse *r_2_* relaxivity and SAR. Therefore, 20 nm NCs were selected for realizing ANCs coated either with a double OA layer or with BSA. Moreover, the BSA-ANCs exhibited the longest stability under physiological conditions, with an average hydrodynamic diameter of about 100 nm, stable for at least seven days. No significant toxicity was detected in J774 murine macrophages or U87-MG tumor cells exposed to the ANCs.

In vivo, the BSA-ANCs showed significant tumor accumulation at 24 h after tail vein injection, both in the presence and absence of an external magnet next to the malignant site. The diseased tissue was readily identified with both NIRF imaging (upon labeling ANCs with Cy5.5 dye) and MRI. Notably, tumor progression was clearly reduced by exposing mice to an AMF for 30 min a day for three consecutive days following the systemic injection of BSA-ANCs.

The superior MRI and magnetic heating properties achieved with one NC configuration and the favorable stability and cytotoxicity profiles suggest that the entrapment of NCs in a single assembly is a valuable strategy for cancer detection and therapy.

## 4. Materials and Methods

### 4.1. Chemicals

Iron (III) acetylacetonate (99%), oleic acid (technical grade 90%), benzyl ether (98%), and BSA (98%) were purchased from Sigma-Aldrich (St. Louis, MO, USA) and used as received; 4-biphenylcarboxylic acid (99%, Acros Organics, Belgium, WI, USA), acetone (99.5%), hexanes (98.5%), sodium bicarbonate (99.7%), nitric acid (70%), and hydrogen peroxide (30%) were purchased from Fisher Scientific (Waltham, MA, USA). The BSA Cy5.5 conjugate (BSA Cy5.5, 10 mg·mL^‒1^ in saline) was purchased from Protein Mods (Burlington, ON, Canada).

### 4.2. Synthesis of Iron Oxide Nanocubes

The synthesis of iron oxide NCs was modified from a previously reported protocol [20]. Briefly, iron (III) acetylacetonate (0.53 g, 1.5 mmol) was mixed with OA (1.27 g, 4.5 mmol), 4-biphenylcarboxylic acid (0.4 g, 2 mmol), and benzyl ether (20 g, 150 mmol). The mixture was heated to 60 °C for 1 h and further heated to 200 °C for 2 h. After the temperature was increased to 280 °C at the rate of 20 °C·min^‒1^, the reaction mixture was maintained at this temperature for 1 h under N_2_. Several NCs ranging in size from 11 to 26 nm were synthesized by changing the amount of iron (III) acetylacetonate (up to 0.9 g, 2.5 mmol) or, benzyl ether (down to 10 g), or reflux time (up to 2 h at 280 °C). The resulting black colloidal solution was purified by adding 35 mL acetone and was centrifuged at 4150 rpm for 30 min. The black precipitated pellets were re-dispersed using hexanes. The purification was repeated three to five times. Finally, NCs of different sizes were prepared and stored in hexanes/toluene.

### 4.3. Double OA-Coated ANCs

The method for coating NCs with a double oleic acid (dOA) layer was a modification of a previously published procedure [26]. Briefly, OA (1 to 10 µM) was mixed with 1 mL of a solution of NCs dispersed in hexanes (1500–4000 mg·L^‒1^ of iron concentration) and 10 mL of sodium bicarbonate buffer (0.1 M). Two organic/aqueous layers of a sample were probe-sonicated (UP 50 H, Dr. Hielscher, Ringwood, NJ, USA) at 60% amplitude for 3 min. The resulting solution was stirred further and stored uncovered for one day to evaporate the residual organic solvents. Finally, the remaining solution was purified using ultracentrifugation (Optima L-90K Ultracentrifuge, Beckman Coulter, Brea, CA, USA) at 35,000 rpm for 2 h, followed by syringe filtration (pore size of 0.45 µm, Whatman NYL, Florham Park, NJ, USA).

### 4.4. BSA-Coated ANCs

For the BSA-ANCs, 3 mL BSA solution (5 mg·mL^‒1^) was mixed with 1 mL of a solution of NCs dispersed in hexanes (1000–3000 mg·L^‒1^) and 10 mL sodium bicarbonate buffer (0.1 M). The reaction mixture was probe-sonicated (UP 50 H, Dr. Hielscher, Ringwood, NJ, USA) at 60% amplitude for 1–2 min in an ice bath. The mixing solution was further stirred for one day without covering to obtain a clear solution (BSA-ANCs) and purified by two rounds of ultracentrifugation (Optima L-90K Ultracentrifuge, Beckman Coulter) at 35,000 rpm for one hour per repetition, followed by syringe filtration (pore size of 0.45 µm, Whatman NYL).

### 4.5. Cell Cultures

U87 and J774 cells from ATCC were cultured in Eagle’s minimum essential medium and Dulbecco’s modified Eagle’s medium (Caisson Lab, Smithfield, NC, USA), respectively, supplemented with 10% fetal bovine serum and 1% penicillin/streptomycin (Thermo Scientific, Waltham, MA, USA). The cells were incubated in a humidified atmosphere with 5% CO_2_ at 37 °C.

### 4.6. Cell Proliferation Assay (MTT Assay)

Cell viability was determined with the MTT cell proliferation assay kit (Promega, Madison, WI, USA) after incubation with ANCs. Respectively, 15,000 U87 cells and 20,000 J774 cells were plated in each well of a 96-well plate. After 24 h, dOA-ANCs and BSA-ANCs were added to the cell medium at different iron concentrations. After 48 h of incubation, the MTT assay was performed according to the manufacturer’s instructions, and the absorbance was measured with a microplate reader (SynergyH4, Bioteck, Winooski, VT, USA).

### 4.7. Cellular Uptake of ANCs

U87 and J774 cells were incubated with dOA-ANCs or BSA-ANCs at different iron concentrations (0.5, 1, 5, 10, 20, 50, and 100 ppm) and visualized during the incubation. For each condition, different zones were randomly chosen and photographed after 24 h of incubation with the ANCs using a phase-contrast inverted microscope (Nikon Eclipse Ti, Melville, NY, USA) with a digital camera (Andor Technology LUCAS, Belfast, UK).

### 4.8. Tumor Model

For the tumor model, 10^6^ U87 cells in 100 µL PBS were injected into the flanks of 12-week-old female nude mice (athymic nude) purchased from Charles River (Boston, MA, USA). Mice were kept on a 12 h light-dark cycle with food and water. All animal experiments in this study were approved by the Institutional Animal Care & Use Committee of Houston Methodist Research Institute.

### 4.9. In Vivo Optical Imaging and Biodistribution

At 10–15 days after tumor implantation, the animals were used for in vivo experiments. Before administration of the ANC suspension and all of the in vivo imaging procedures, the mice were anesthetized with isoflurane and kept under its influence for the injection and the duration of the experiment. A suspension of 20 nm ANCs coated with Cy5.5-BSA (250 µg_Fe_·kg^‒1^ body weight, 100 µg Cy5.5-BSA·kg^‒1^ body weight) in saline was injected intravenously through the tail vein using a 0.5-mL insulin syringe. During and after injection of the ANC suspension, a handheld magnet (D401-N52, K&J Magnetics, Inc., Pipersville, PA, USA) was placed on top of each tumor for 40 min. After 24 h, the mice (*n* = 3 in each group) were imaged using a Caliper IVIS-200 in vivo bioluminescence/fluorescence imaging system (PerkinElmer, Waltham, MA, USA) with an F-stop of 2 and an exposure of one second. The final fluorescent images were generated with an optimal excitation filter of 680 nm and an emission filter of 700 nm. The organs (lung, heart, liver, spleen, kidney, and tumor) were collected from the sacrificed mice and imaged with an IVIS-200 to evaluate the biodistribution.

### 4.10. In Vivo MR Imaging

Mice (*n* = 3) were injected intravenously with 20 nm BSA-ANCs (5 mg_Fe_·kg^‒1^ body weight) in saline through the tail vein, while a handheld magnet was placed on top of each tumor for 40 min. MR images were acquired at 4 and 24 h p.i. using a wrist coil. T_2_-weighted MR images were acquired in a 3T clinical scanner (Philips Ingenia) using a spin echo sequence with TR = 3000 ms, TE = 100 ms, and a slice thickness of 500 µm. The field of view was 80 × 80, and the reconstructed resolution matrix was 512 × 512.

### 4.11. In Vivo Magnetic Hyperthermia Treatment

For the hyperthermia trials, a suspension of 20 nm BSA-ANCs in saline (5 mg_Fe_·kg^‒1^ body weight) was intravenously administrated through the tail vein (*n* = 3), and a handheld magnet was placed on top of the tumor for 40 min during and after the injection. After 24 h, magnetic hyperthermia was carried out for 30 min using a custom-built radio-frequency generation system. The AMF was generated at a frequency of 512 KHz and a field amplitude of 10 kA·m^‒1^. The mapping of the temperature was monitored using a FLIR A325 infrared camera (FLIR Systems, Inc., Wilsonville, OR, USA). After three days of treatment, the tumor growth was monitored daily as tumor volume, calculated as 0.5 × (major axis)^2^ × (minor axis).

## Supplementary Materials

The following are available online at http:www.mdpi.com/2079-4991/7/4/72/s1 Figures S1–S9.
