# Supplementary material for: Assembly of Iron Oxide Nanocubes for Enhanced Cancer Hyperthermia and Magnetic Resonance Imaging
Source: Nanomaterials (Basel). 2017 Mar 28;7(4):72. doi: 10.3390/nano7040072 (PMC5408164; doi:10.3390/nano7040072)
Supplement: Supplementary file 1 [file nanomaterials-07-00072-s001.pdf]

Supplementary Information

Assembly of Iron Oxide Nanocubes for Enhanced Cancer Hyperthermia and Magnetic Resonance Imaging

Minjung Cho [1,2], Antonio Cervadoro [6], Maricela R. Ramirez [1], Cinzia Stigliano [1], Audrius Brazdeikis [3], Vicki L. Colvin [2], Pierluigi Civera [5,+], Jaehong Key [4,*], and Paolo Decuzzi [6,*]

[1] Department of Translational Imaging & Department of Nanomedicine, Houston Methodist Research Institute, Houston, TX 77030, USA
[2] Department of Chemistry, Rice University, Houston TX 77005, USA
[3] Department of Physics and Texas Center for Superconductivity, University of Houston, Houston TX 77004, USA
[4] Department of Biomedical Engineering, Yonsei University, 1 Yonseidae-gil, Wonju, Gangwon-do, 220-710, South Korea
[5] Dipartimento di Elettronica e Telecomunicazioni, Politecnico di Torino, I-10129 Torino, Italy
[6] Laboratory of Nanotechnology for Precision Medicine, Fondazione Istituto Italiano di Tecnologia, Genova, 16163, Italy

[+] Pierluigi Civera died on October 28, 2014.
* Correspondence: jkey@yonsei.ac.kr; Tel.: +82-10-5315-0626 (J.K.); Paolo.Decuzzi@iit.it; Tel.: 39-010-71781-941 (P.D.)

Synthesis of iron oxide nanocubes (individual NCs). This synthesis was performed according to the method in our previous study [1]. Briefly, FeCl3·6H2O (5.4 g, 20 mmol), sodium oleate (18.5 g, 62 mmol), ethanol (40 ml), deionized water (30 ml), and hexane (70 ml) were mixed and heated to 60 °C for 4 hours in a refluxing system under N_2 flow.[2] The resulting organic phase containing iron oleate was washed with deionized water and then purified with acetone through three rounds of centrifugation (11,000 rpm, 30 minutes). For individual 11 nm iron oxide NCs, iron oleate (0.6 g, 0.1 mmol), sodium oleate (0.3 g, 1 mmol), oleic acid (0.56 g, 2 mmol), and 1-ODE (5 g, 20 mmol) were mixed in a 50 ml three-neck flask and heated to 315 °C for 1 hour under N_2. Individual 15 nm iron oxide NCs were synthesized by changing the amounts of iron oleate precursors (1.8 g, 0.3 mmol), and oleic acid (0.56 g, 2 mmol), and increasing the reflux time (2 hours at 315 °C). To prepare 20 nm and 28 nm iron oxide NCs, FeOOH (0.36 g, 4 mmol), oleic acid (16 mmol (4.52 g) or 20 mmol (5.7g)), sodium oleate (0.9 g, 3 mmol), and 1-ODE (10 g, 40 mmol) were mixed and heated to 120 °C for 6 hours to remove residual water and continuously heated to 240°C for 0.5 hours for the synthesis of iron oleate precursor. The reaction mixture was further heated to 315°C for 3 hours under N_2.

Transmission electron microscopy (TEM). The TEM images were produced by a JEOL 2,100 field emission gun TEM operating at 200 kV with a single tilt holder using ultrathin carbon type-A 400 mesh copper grids (Ted Pella Inc.). The average sizes and size variations were obtained by counting more than 1,000 particles using Image-Pro Plus 5.0 (Media Cybernetics, Inc., Silver Spring, MD).

Dynamic light scattering (DLS) and zeta potential analysis. All water-soluble ANC suspensions were analyzed by DLS and zeta potential to measure average hydrodynamic size (nm) and surface charge (mV), respectively, using a ZEN-3,600 Zetasizer Nano (Malvern, UK) equipped with a HeNe 633 nm laser. The average hydrodynamic size (nm) was calculated as the mean size of the first peak in the number distribution. Standard deviations of hydrodynamic size (nm) and zeta potential (mV) were calculated from five replicate measurements.

Inductively coupled plasma optical emission spectroscopy (ICP-OES). The concentration of iron in ANC suspension was measured by a Perkin Elmer ICP-AES instrument equipped with an auto sampler. Purified ANC solutions were acid digested using HNO_3 (70 %) and H_2O_2 (30 %) and diluted with deionized water for ICP analysis.

Magnetic resonance relaxivity measurement. To measure the relaxivities r_1 and r_2 of ANCs, a magnetic resonance relaxometer was used at 1.41 T (NMR analyzer, mq 60, Bruker). Various concentrations of ANC solution were prepared (0.014 ÷ 0.35 mM for r_1 and 0.0014 ÷ 0.022 mM for r_2), and the relaxivity values were calculated by the ratio of $1/T_{1,2}$ and concentration (mM) of Fe. The r_1 and r_2 were calculated using **Equation 1** [3]:

$$r_{1,2} = \left(\frac{1}{T_{1,2}} - \frac{1}{T_{water}}\right)/[Fe] \qquad (1)$$

where $T_{1,2}$ represents the relaxation time of the sample, T_{water} is that for water, $r_{1,2}$ (mM^{-1} s^{-1}) are the relaxivity values, and [Fe] is the concentration of Fe.

Specific absorption rate measurements for hyperthermia treatment. The magnetic heating property of ANCs was measured by a custom-built hyperthermia system. The measurement was performed using a radio frequency generator producing an alternating magnetic field (AMF) with a frequency (f) of 512 KHz and field amplitude (H) of 10 kA m^{-1} [4]. A cooling system thermally isolated the vial from the high temperature of the coil when applying AMF. ANC suspension (600 µL) was placed in a cylindrical probe (4 mm ID x 40 mm height), and the temperature was monitored with an optical probe (OptiSens Instrument) immersed into the geometrical center of the solution. When the sample suspension reached the equilibrium temperature (~ 19 °C), the magnetic field was activated, and the temperature was recorded every second for about 15-20 minutes. The specific absorption rate (SAR) was calculated based on **Equation 2** [5]:

$$SAR = \left.\frac{\Delta T}{\Delta t}\right|_{t=0} C_p \frac{1}{m_{Fe}} \qquad (2)$$

where T is the temperature of the nanocube suspension, t is the time, C_p is the heat capacity of the buffer, and m_{Fe} is the final mass fraction of the iron in the sample suspension.

Superconducting Quantum Interference Device characterization (SQUID). To determine the magnetic behavior and saturated magnetization value of iron oxide NCs, the magnetization measurement was carried out with a Quantum Design SQUID magnetometer MPMS-XL. The sample was prepared by injecting a certain amount of nanocube suspension (300 µL, 3,000~5,000 mg L^{-1}) into an NMR tube. The tube was then sealed under vacuum and placed into a plastic straw for measurements. DC magnetic measurement was performed at 300 K in a 0-50 Oe field. Magnetization and hysteresis curves were collected in the range from -50 KOe to 50 KOe at 300 K.

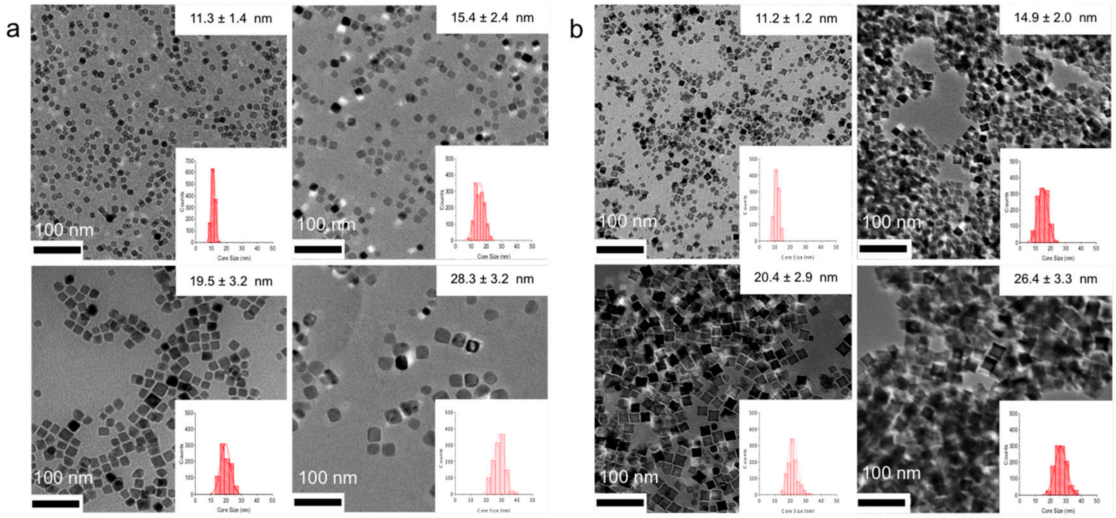

Precursor	Surfactant	Solvent	Temperature/Time	Heat rate (°C/min)	NCs diameter	Result
Iron oleate or FeOOH	Sodium oleate, Oleic acid	ODE	315°C (1h) or 120°C (6h), 240°C (0.5h), 315°C (2h)	20°C/min	11~28 nm	Individual NCs
$Fe(acac)_3$	Oleic acid, 4-byphenylcarboxylic acid	Benzyl ether	60°C (1h), 200°C (2h), 280°C (0.5h-3h)	20°C/min	11~26 nm	Assembled NCs

Supplementary Figure 1. (a) TEM images of individual NCs in hexane: the core sizes of individual NCs were 11.3 ± 1.4 nm, 15.4 ± 2.4 nm, 19.5 ± 3.2 nm, and 28.3 ± 3.2 nm. (b) TEM images of assembled NCs in hexane: the core sizes of clustered NCs were 11.2 ± 1.2 nm, 14.9 ± 2.0 nm, 20.4 ± 2.9 nm, and 26.4 ± 3.3 nm. Insets provide the size distribution for each NC configuration. (Scale bar: 100 nm). The table summarizes the experimental conditions for the synthesis of individual and assembled NCs, specifying the iron source, surfactant, solvent, and temperature (see the Methods section).

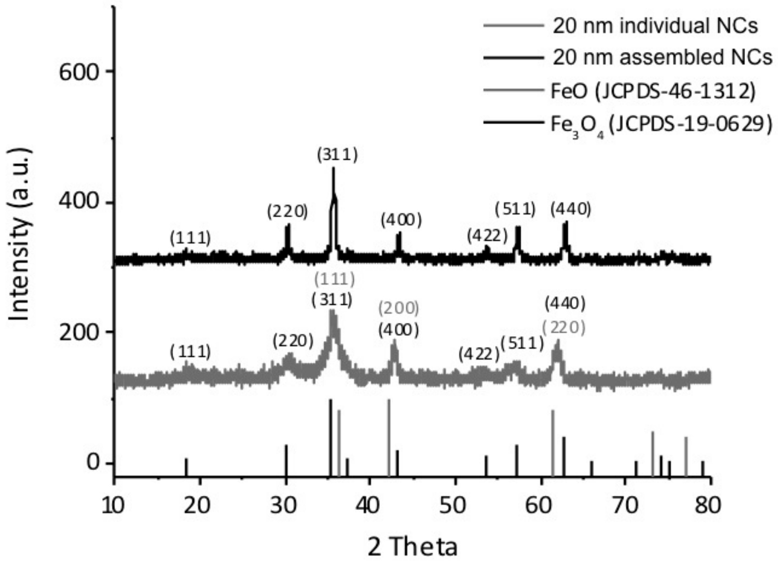

Supplementary Figure 2. X-ray diffraction patterns of individual and assembled NCs compared to the references materials magnetite (Fe_3O_4) and wüstite (FeO)

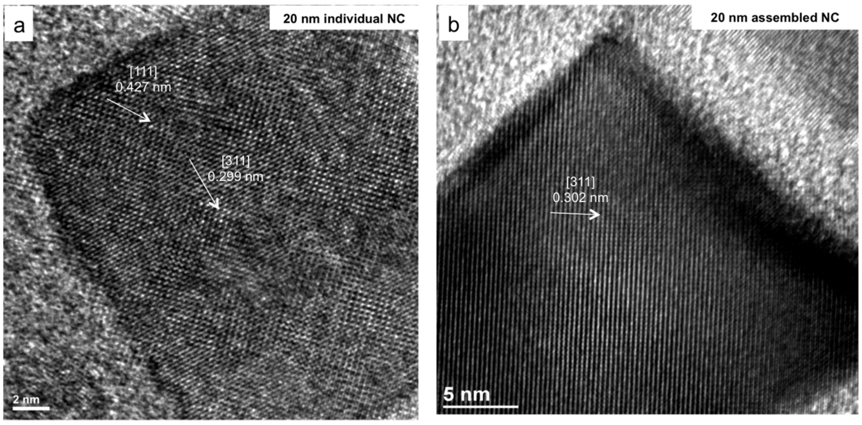

Supplementary Figure 3. High-resolution TEM (HR-TEM) images of individual and assembled NCs, showing the crystalline lattice fringes.

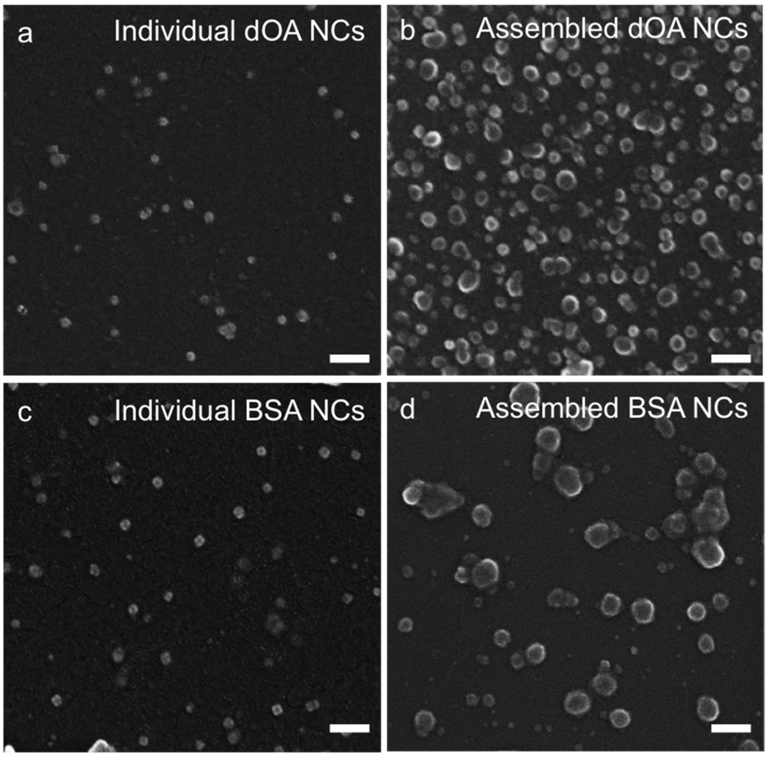

Supplementary Figure 4. Scanning electron microscopy images (SEM) of 20 nm dOA-coated individual and assembled iron oxide nanocubes (NCs) in water (a and b), and BSA-coated individual and assembled iron oxide NCs (c and d) in water. The scale bars are 200 nm.

a

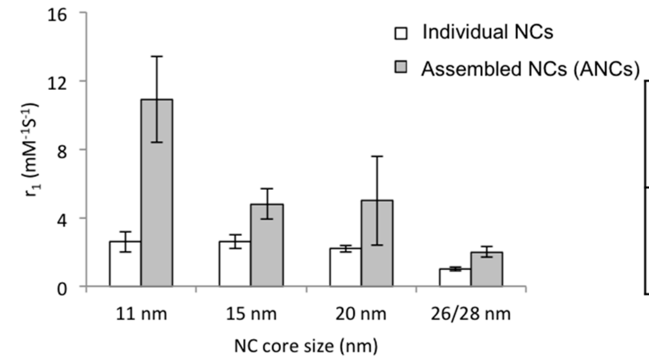

	core size (nm)	r_1(mM^{-1}S^{-1})
Individual NCs	11	2.6 ± 0.6
	15	2.6 ± 0.4
	20	2.2 ± 0.2
	28	1.0 ± 0.1
Assembled NCs	11	10.9 ± 2.5
	15	4.8 ± 0.9
	**20**	**5.0 ± 2.6**
	26	2.0 ± 0.3

b

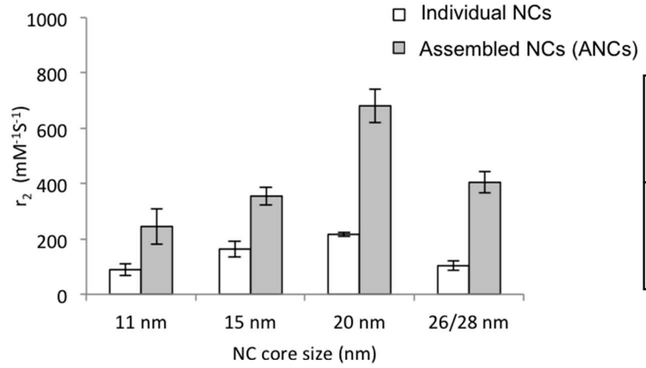

	core size (nm)	r_2(mM^{-1}S^{-1})
Individual NCs	11	88.6 ± 20.3
	15	163.3 ± 28.1
	20	215.5 ± 7.4
	28	103.8 ± 16.9
Assembled NCs	11	244.1 ± 64.0
	15	354.2 ± 31.1
	**20**	**678.9 ± 29.0**
	26	405.7 ± 38.7

c

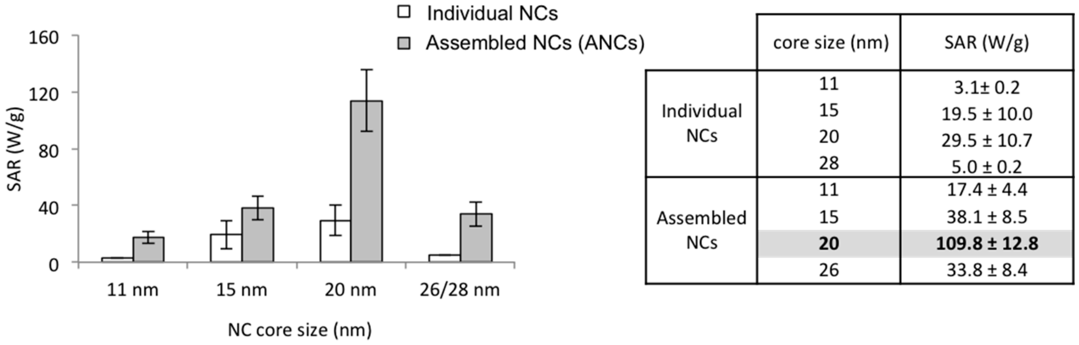

	core size (nm)	SAR (W/g)
Individual NCs	11	3.1± 0.2
	15	19.5 ± 10.0
	20	29.5 ± 10.7
	28	5.0 ± 0.2
Assembled NCs	11	17.4 ± 4.4
	15	38.1 ± 8.5
	**20**	**109.8 ± 12.8**
	26	33.8 ± 8.4

Supplementary Figure 5. MRI relaxivity and magnetic heating efficiency of NCs. a) Longitudinal relaxivity r_1 of individual and assembled NCs; b) Transverse relaxivity r_2 of individual and assembled NCs; c) specific absorption rate (SAR) of individual and assembled NCs. The table lists the average r_1, r_2, and SAR values with standard deviation.

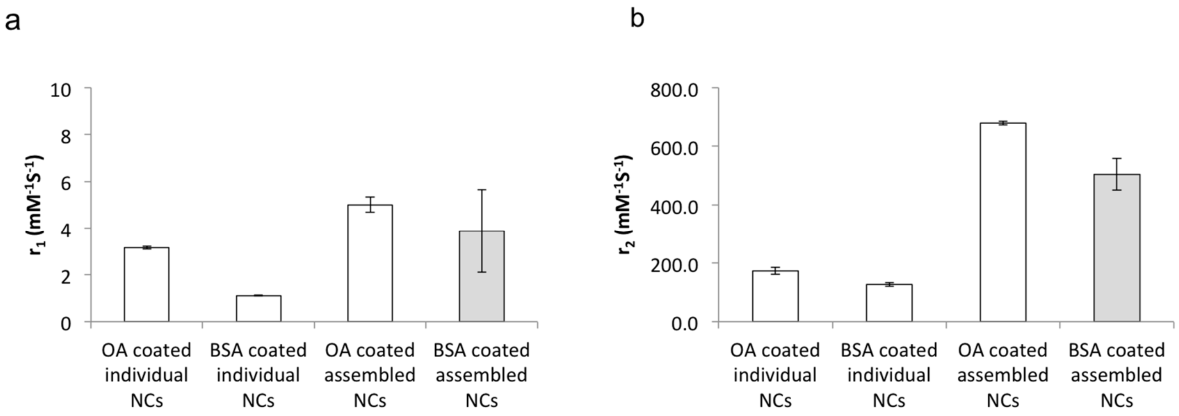

	core size (nm)	$r_1 (mM^{-1}S^{-1})$
dOA coated individual NCs	20	3.2 ± 0.1
BSA coated individual NCs	20	1.1 ± 0.03
dOA coated assembled NCs	20	5.0 ± 0.3
BSA coated assembled NCs	20	3.9 ± 1.8

	core size (nm)	$r_2 (mM^{-1}S^{-1})$
dOA coated individual NCs	20	173.1 ± 11.5
BSA coated individual NCs	20	126.9 ± 6.8
dOA coated assembled NCs	20	678.9 ± 2.9
BSA coated assembled NCs	20	504.2± 53.9

Supplementary Figure 6. MRI relaxivity values of 20 nm individual and assembled NCs with different surface coatings: (a) r_1 values of individual and assembled NCs with dOA and BSA coatings; (b) r_2 values of individual and assembled NCs with dOA and BSA coatings.

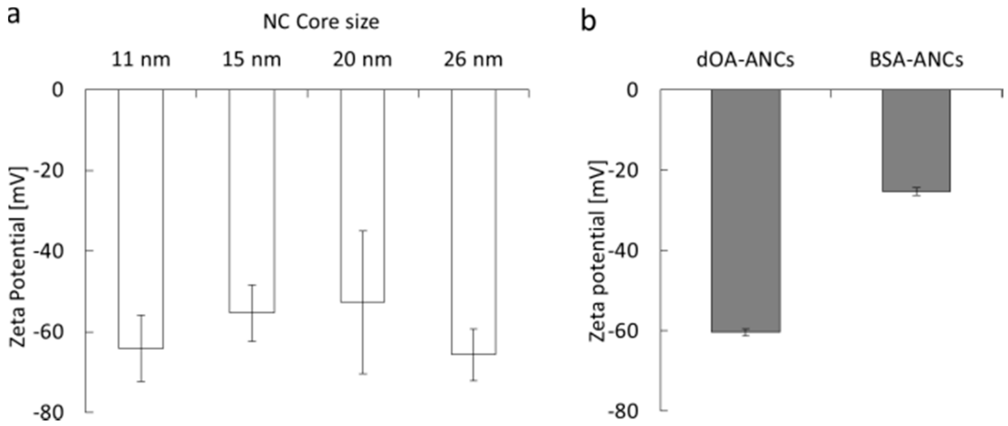

Supplementary Figure 7. Zeta potential of the assembled NCs. (**a**) Zeta potential values of dOA-ANCs with different edge lengths (11-26 nm); (**b**) Zeta potential values of 20 nm dOA-ANCs and BSA-ANCs.

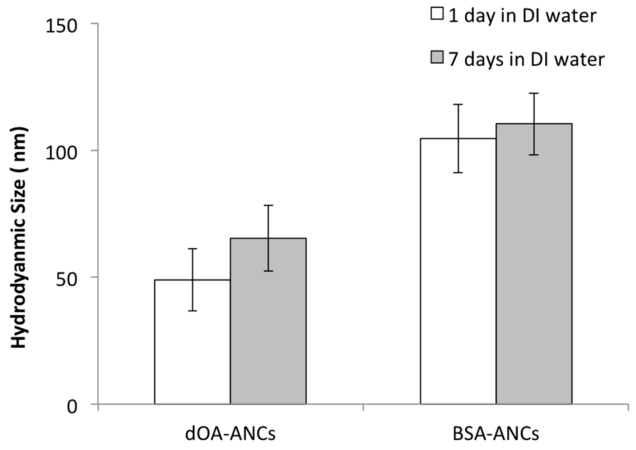

	1 day in DI Water	7 days in DI water
dOA-ANCs	48.79 ± 12.19 nm	65.23 ± 13.12 nm
BSA-ANCs	104.55 ± 13.51 nm	110.32 ± 12.21 nm

Supplementary Figure 8. The hydrodynamic diameter stability in deionized water of 20 nm assembled NCs (ANCs), coated with dOA or BSA at 1 and 7 days.

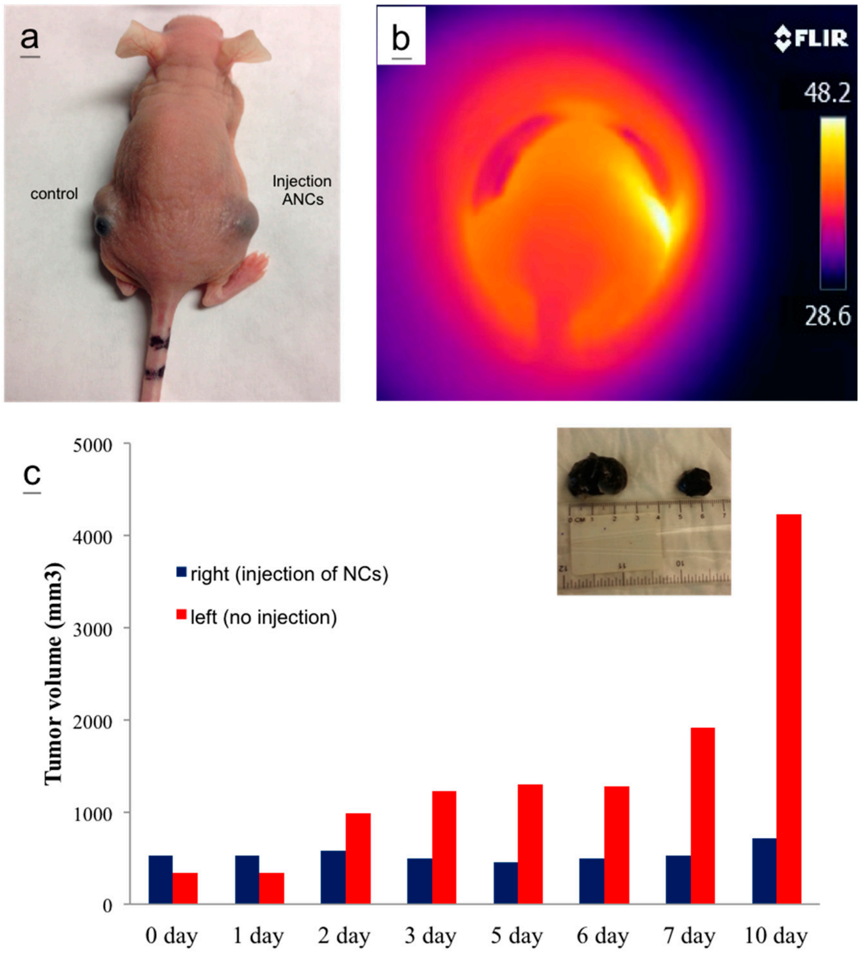

Supplementary Figure 9. *In vivo* magnetic hyperthermia experiment. (a) Animal model with two U-87-MG tumors (left: no injection, right: systemic injection of 5 mg/kg of BAS-ANCs). An external magnet was attached to the right tumor for 40 min during the injection period. (b) Temperature map of 30 min post treatment (the mice lying within the coil). The right tumor area with injection of ANCs showed a higher temperature (38-42 °C) in the bright image. (c)

Monitoring of tumor volume for 10 days. The left tumor grew quickly compared to the right tumor with ANC injection. The tumor growth was monitored daily as tumor volume (0.5 x (major axis)^2 x (minor axis).

References

1. Yu, W.W., et al., *Synthesis of monodisperse iron oxide nanocrystals by thermal decomposition of iron carboxylate salts.* Chem Commun (Camb), 2004(20): p. 2306-7.
2. Park, J., et al., *Ultra-large-scale syntheses of monodisperse nanocrystals.* Nature Materials, 2004. **3**(12): p. 891-895.
3. Lee, N., et al., *Water-dispersible ferrimagnetic iron oxide nanocubes with extremely high r(2) relaxivity for highly sensitive in vivo MRI of tumors.* Nano Lett, 2012. **12**(6): p. 3127-31.
4. Cervadoro, A., et al., *Design maps for the hyperthermic treatment of tumors with superparamagnetic nanoparticles.* PLoS One, 2013. **8**(2): p. e57332.
5. Guardia, P., et al., *Water-soluble iron oxide nanocubes with high values of specific absorption rate for cancer cell hyperthermia treatment.* ACS Nano, 2012. **6**(4): p. 3080-91.
